# Detection of a microbial metabolite by STING regulates inflammasome activation in response to *Chlamydia trachomatis* infection

**DOI:** 10.1371/journal.ppat.1006383

**Published:** 2017-06-01

**Authors:** Steve J. Webster, Sven Brode, Lou Ellis, Timothy J. Fitzmaurice, Matthew J. Elder, Nelson O. Gekara, Panagiotis Tourlomousis, Clare Bryant, Simon Clare, Ronnie Chee, Hill J. S. Gaston, Jane C. Goodall

**Affiliations:** 1 Rheumatology Research Group, Department of Medicine, University of Cambridge, Cambridge, United Kingdom; 2 Molecular Infection Medicine Sweden, Umeå Centre for Microbial Research, Department of Molecular Biology, Umeå University, Umeå, Sweden; 3 Department of Veterinary Medicine, University of Cambridge, Cambridge, United Kingdom; 4 Wellcome Trust Sanger Institute, Wellcome Trust Genome Campus, Hinxton, United Kingdom; 5 Department of Immunology, Royal Free Hospital, London, United Kingdom; University of São Paulo FMRP/USP, BRAZIL

## Abstract

The innate immune system is a critical component of host defence against microbial pathogens, but effective responses require an ability to distinguish between infectious and non-infectious insult to prevent inappropriate inflammation. Using the important obligate intracellular human pathogen *Chlamydia trachomatis;* an organism that causes significant immunopathology, we sought to determine critical host and pathogen factors that contribute to the induction of inflammasome activation. We assayed inflammasome activation by immunoblotting and ELISA to detect IL-1β processing and LDH release to determine pyroptosis. Using primary murine bone marrow derived macrophages or human monocyte derived dendritic cells, infected with live or attenuated *Chlamydia trachomatis* we report that the live organism activates both canonical and non-canonical inflammasomes, but only canonical inflammasomes controlled IL-1β processing which preceded pyroptosis. NADPH oxidase deficient macrophages were permissive to *Chlamydia trachomatis* replication and displayed elevated type-1 interferon and inflammasome activation. Conversely, attenuated, non-replicating *Chlamydia trachomatis*, primed but did not activate inflammasomes and stimulated reduced type-1 interferon responses. This suggested bacterial replication or metabolism as important factors that determine interferon responses and inflammasome activation. We identified STING but not cGAS as a central mediator of interferon regulated inflammasome activation. Interestingly, exogenous delivery of a *Chlamydia trachomatis* metabolite and STING ligand—cyclic di-AMP, recovered inflammasome activation to attenuated bacteria in a STING dependent manner thus indicating that a bacterial metabolite is a key factor initiating inflammasome activation through STING, independent of cGAS. These data suggest a potential mechanism of how the innate immune system can distinguish between infectious and non-infectious insult and instigate appropriate immune responses that could be therapeutically targeted.

## Author summary

Innate responses to bacterial infection such as *Chlamydia trachomatis* activate inflammasomes to enable the processing of IL-1β, IL-18 and the induction of an inflammatory form of cell death termed pyroptosis. Inflammasomes are crucial to host defence but require tight regulation in order to prevent inappropriate inflammation and immunopathology. Here, we demonstrate that the pro-inflammatory potential of an attenuated strain of *Chlamydia trachomatis*, that fails to activate the inflammasome, can be rescued by the addition of a bacterial metabolite. The requirement for this metabolite, highlights a novel mechanism of inflammasome regulation and reveals a crucial role for STING mediated interferon signalling independent of cGAS. These findings further our understanding of how the innate immune system can differentiate between potential infectious and non-infectious threats and mount appropriate immune responses.

## Introduction

The obligate intracellular pathogen *Chlamydia trachomatis* is a major cause of infectious disease world-wide and can initiate inflammatory pathology such as pelvic inflammatory disease, reactive arthritis and infectious blindness (trachoma). Significantly, murine models of *Chlamydia* infection demonstrate that host inflammatory mediators, particularly the inflammatory cytokine IL-1β, type-1 interferons, caspase-1 and caspase-11 account for a significant proportion of infection associated pathology [1–3].

Inflammasomes are molecular scaffolds that facilitate the activation of inflammatory caspases resulting in the proteolytic processing of the cytokines IL-1β and IL-18 in addition to the induction of a form of programmed necrosis termed pyroptosis [4, 5]. Recently, a non-canonical inflammasome requiring caspase-11 (caspase-4/5 in humans) has been identified that responds specifically to LPS contamination of the cytosol, independent of TLR4 [6–9]. Uniquely, activation of the non-canonical inflammasome does not require an upstream sensor that is required for canonical caspase-1 activation, and occurs as a consequence of LPS being directly recognised by Caspase-11 (or Caspase-4/5) [10] and is critically dependent on the acylation status of the lipid-A moiety [9]. The non-canonical inflammasome is essential for pyroptosis and IL-1β maturation in response to infection with certain gram-negative bacterial pathogens, or the delivery of cytoplasmic LPS, and occurs as a consequence of the caspase-11 dependent cleavage of gasdermin-D (GSDMD) [11, 12] and pannexin-1 [13]. Activation of the non-canonical inflammasome in response to bacterial pathogens can occur as a direct result of cytosolic invasion by bacteria that occurs during infection with *Burkolderia thailandensis*, resulting in a rapid execution of caspase-11 mediated pyroptosis, essential to host defence [14]. Alternatively, non-canonical inflammasome activation can occur as a consequence of LPS being released from bacterial pathogen containing vacuoles (PV), via the action of Immune Related GTPases (IRG’s) and Guanylate Binding Proteins (GBP’s) [15].

Despite type-1 interferons being reported to inhibit canonical inflammasome activation [16], non-canonical inflammasomes require interferon signalling for up-regulation of caspase-11 [17], GBP and IRG expression [18].

Mechanisms controlling inflammasome activation in response to *Chlamydia sp* infection are still poorly understood, although NLRP3, reactive oxygen species, mitochondrial damage and oxidised mitochondrial DNA have been implicated in the process [19, 20] [21, 22]. In addition, recent work using interferon-primed macrophages has demonstrated that GBP’s, NLRP3 and AIM2 participate in IL-18 maturation in response to *Chlamydia trachomatis* infection, while cell death occurs via either caspase-1 or caspase-11 activity, suggesting engagement of the non-canonical inflammasome [23]. Importantly, *Chlamydia muridarum* infection of macrophages induces type-1 interferon expression via Stimulator of Interferon Gene (STING) activity, implying that *Chlamydia sp* infections of macrophages are ‘self-priming’ and exogenous interferon is not necessary [24]. However, mechanisms controlling this process are poorly understood.

Here, we report that inflammasome activation in un-primed bone marrow derived macrophages (BMDM) required replication or metabolic activity of *Chlamydia trachomatis*. We also provide evidence that production of a chlamydial metabolite, but not detection of host or microbial DNA by cGAS, activated STING initiating autocrine, type-1 interferon signalling leading to canonical and non-canonical inflammasome activation. We propose that intracellular *Chlamydia trachomatis* replication or metabolism, and the subsequent detection of cyclic di-AMP by STING, are key events initiating inflammasome activation in response to *Chlamydia trachomatis* infection.

## Results

### Cell death and IL-1β maturation in response to *C*. *trachomatis* infection occur asynchronously and are regulated through different inflammasomes

**Fig 1:**
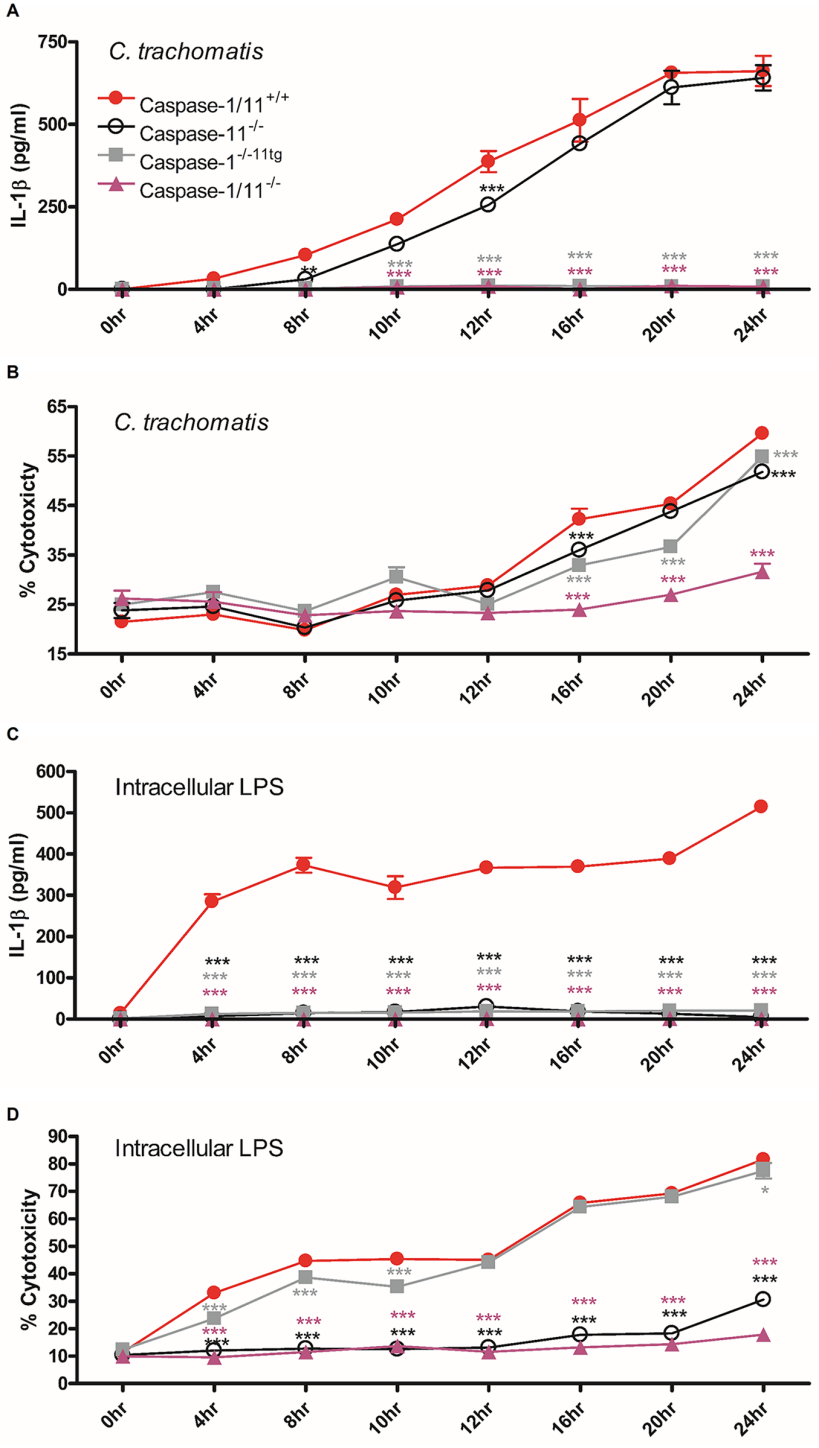
IL-1β maturation is independent of caspase-11 and precedes pyroptosis which is regulated by both canonical and non-canonical inflammasomes in response to *C*. *trachomatis* infection. Time course of IL-1β secretion analysed by ELISA or cell death measured by LDH release in supernatants from *C*. *trachomatis* infected (A+B) or LPS transfected (C+D) BMDM from wild-type (Caspase-1/11^+/+^), caspase-11 knock-out (Caspase-11^-/-^), caspase-1 knock-out caspase-11 transgenic (Caspase-1^-/-11tg^) or caspase-1/caspase-11 knock-out (Caspase-1/11^-/-^). Data represent the mean values from BMDM obtained from individual mice, performed in biological triplicate and are representative of 2-similar experiments. Error bars indicate ±SEM, *p = <0.05, **p = <0.01 and ***p = <0.001 vs Caspase-1/11^+/+^.

We sought to identify whether canonical and non-canonical inflammasomes regulate cytokine maturation and cell death in response to *C*. *trachomatis* infection of un-primed BMDM using cytosolic delivery of LPS as a control for non-canonical inflammasome activation. A time course infection of BMDM with *C*. *trachomatis* resulted in significant increases in IL-1β secretion as early as 8–10 hours post infection in wild-type and caspase-11 deficient but not caspase-1 deficient BMDM indicating that the non-canonical inflammasome was not required for IL-1β maturation in response to *C*. *trachomatis* infection (Fig 1A). We next analysed LDH release as an indicator of lytic cell death, associated with pyroptosis and necrosis over the same time course of *C*. *trachomatis* infection (Fig 1B). As expected *C*. *trachomatis* infection induced significant cytotoxicity in wild type BMDM but surprisingly, this was delayed compared to IL-1β release and individual deletion of either caspase-11 or caspase-1 did not confer resistance to cell death suggesting a potential non-pyroptotic cell death mechanism. However, deletion of both caspase-1 and caspase-11 resulted in a dramatic protection of cell death indicating that both inflammatory caspases contribute to the process and the form of cell death is therefore likely to be classical pyroptosis as both caspase-1 and caspase-11 have been demonstrated to cleave and activate the pore forming protein gasdermin D in order to initiate pyroptotic cell death [11, 12]. Intriguingly, despite the ability of both caspase-1 and caspase-11 to induce cell death in response to *C*. *trachomatis* infection, death appeared to be delayed in the caspase-1 ko / caspase-11 transgenic cells, particularly at the 20-hour time point. This suggests that caspase-1 may induce early cell death while caspase-11 contributes later. This is an attractive answer to the conundrum of why caspase-11 does not affect IL-1β but contributes to cell death. Late activity of caspase-11 may not be required for the NLRP3 dependent activation of caspase-1 that controls cytokine maturation, as this may have already occurred through canonical inflammasome activation. We confirmed the functional status of the caspase deficient cells by specific activation of the non-canonical inflammasome using cytosolic delivery of LPS. As expected, intracellular LPS induced IL-1β secretion was dependent on both capase-1 and caspase-11 (Fig 1C) while cell death was only dependent on caspase-11 (Fig 1D) as previously reported [6, 8, 9].

**Fig 2:**
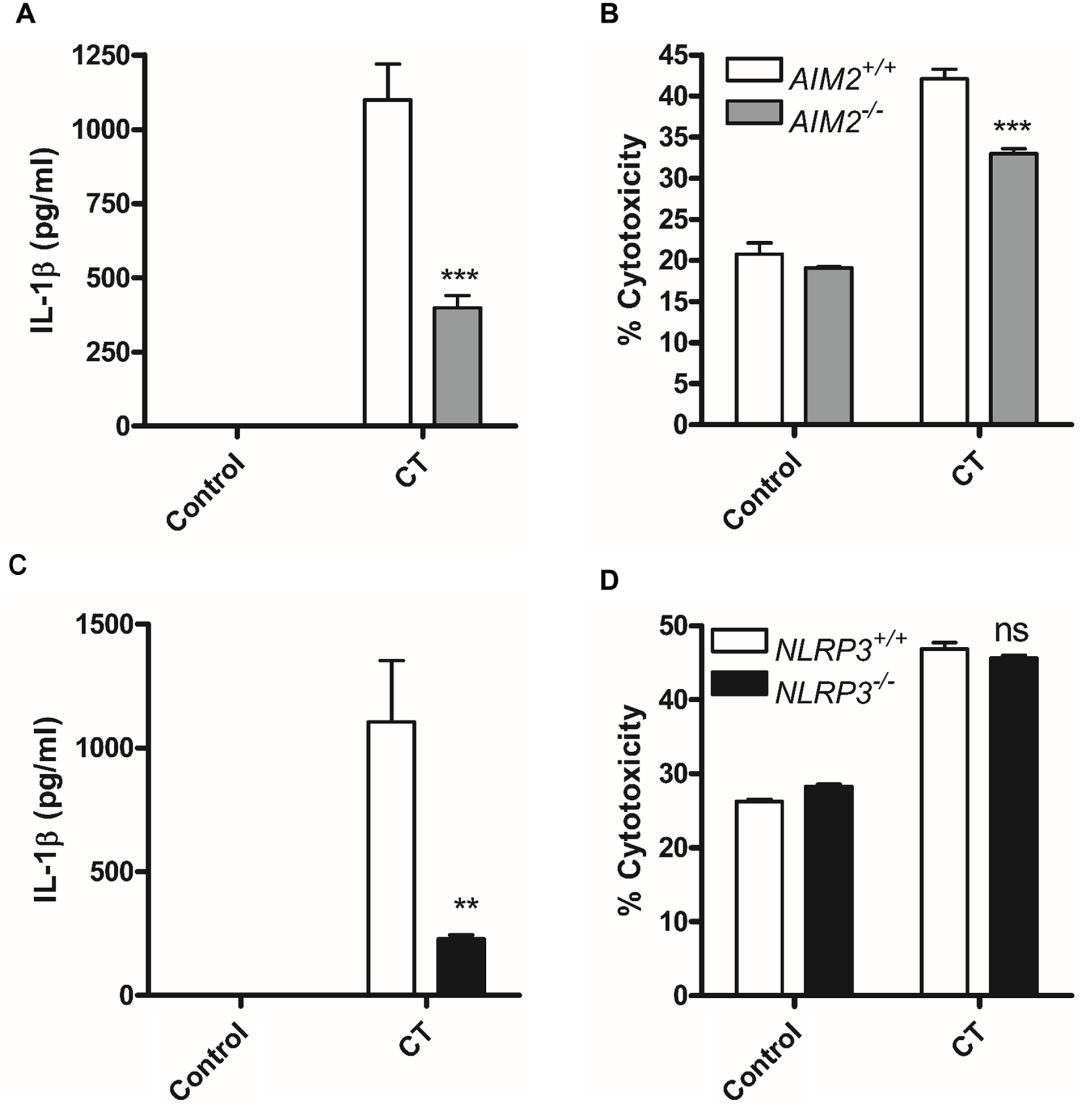
AIM2 and NLRP3 are required for canonical caspase-1 activation in response to *C*. *trachomatis* infection. IL-1β release analysed by ELISA and cell death measured by LDH release following *C*. *trachomatis* infection of wild type (AIM2^+/+^ or NLRP3^+/+^) or AIM2 deficient (AIM2^-/-^) (A + B) or NLRP3 deficient (NLRP3^-/-^) (C + D) BMDM for 24 hours. Data represent the mean values from BMDM obtained from individual mice, performed in biological triplicate. Error bars indicate ±SEM, *p = <0.05, **p = <0.01 and ***p = <0.001, ns indicates no significant difference.

We next examined which canonical intracellular sensor activated caspase-1 in response to *C*. *trachomatis* infection. Both NLRP3 [19, 20] and AIM2 [23] are reported to be activated in response to *C*. *trachomatis* infection enabling caspase-1 activation and the processing of IL-1β. We therefore confirmed the requirement for both inflammasomes in our study (Fig 2A, 2B, 2C and 2D). Both NLRP3 and AIM2 deficient macrophages displayed reduced IL-1β responses to *C*. *trachomatis* infection as expected (Fig 2A and 2C). However, the absence of AIM2 but not NLRP3 had a small but significant effect on cell death in response to infection potentially accounting for the small reduction in cell death we observed with caspase-1 deficiency alone, but also reinforcing our findings that in contrast to IL-1β release, cell death was not solely dependent on caspase-1 activity (Fig 2B and 2D).

Mitochondrial dysfunction is known to contribute to inflammasome activation by sterile agonists [25] and *Chlamydia sp* infection [21, 22]. We therefore investigated whether canonical activation of caspase-1 resulting in IL-1β processing was inhibited by the presence of a mitochondrial anti-oxidant (Mito-Q) (S1A, S1B and S1C Fig). Mito-Q, in a pattern similar to NLRP3 deficiency, inhibited *C*. *trachomatis* induced IL-1β release, but had no effect on cell death, most likely due to the overriding effect of caspase-11 and further supporting our findings that *C*. *trachomatis* induced death could be regulated by both caspase-1 and caspase-11. Importantly, MitoQ did not block pro IL-1β upregulation by *C*. *trachomatis* indicating that MitoQ was blocking caspase-1 mediated IL-1β processing and not priming (S1B Fig). Infection of macrophages with *Brucella abortus* activates the NLRP3 inflammasome in a mechanism requiring IRE-1 mediated ER stress, mitochondrial damage and caspase-2 activity [26]. We have previously demonstrated that *C*. *trachomatis* infection of human dendritic cells results in IRE1α activation [27]. We therefore examined if IRE1α was relevant to *C*. *trachomatis* induced inflammasome activation by utilising IRE-1 deficient macrophages or the IRE-1 inhibitor; 4μ8c. IRE-1 deficient BMDM or BMDM pre-treated with 4μ8c demonstrated normal IL-1β release in response to *C*. *trachomatis* infection (S1D and S1E Fig) indicating that ER stress driven inflammasome activation was not relevant to *C*. *trachomatis* infection and the mitochondrial component differs from that of *B*. *abortus*. Furthermore, our data suggest that canonical activation of caspase-1 through release of DNA (either host or pathogen derived) in to the cytosol activating AIM2 is amplified by mitochondrial ROS to activate NLRP3 and induce maximal inflammasome responses to *C*. *trachomatis*. Both NLRP3 and AIM2 share the requirement for the adaptor ASC to provide a scaffold for the recruitment of caspase-1. The fact that deletion of either AIM2 or NLRP3 only partially attenuated inflammasome responses and that only one ASC speck is formed per cell suggests that maximal inflammasome response to *C*. *trachomatis* requires the co occupation of the ASC filament by both receptors. However, it remains unknown whether recruitment of both receptors occurs simultaneously to initiate the aggregation of the ASC complex resulting in an active inflammasome during infection.

### Attenuated *C*. *trachomatis* induces inflammasome priming but not activation

**Fig 3:**
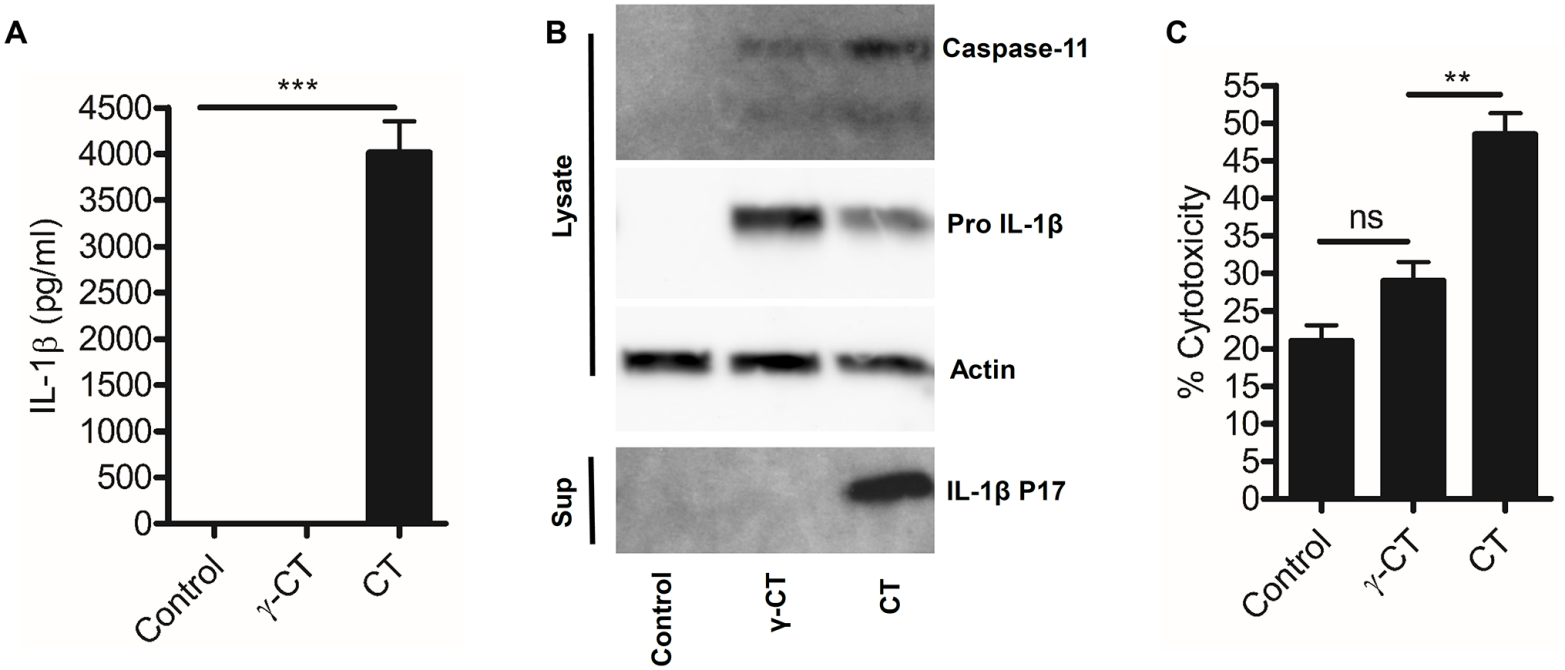
Attenuated *C*. *trachomatis* can prime but not activate inflammasomes. (A) IL-1β secretion analysed by ELISA of supernatants from wild-type BMDM stimulated with attenuated, gamma-irradiated *C*. *trachomatis* particles (γ-CT) or infection with live *C*. *trachomatis* (CT) for 24hrs. Data represent the mean from BMDM obtained from three-individual mice, error bars indicate ±SEM, ***p = <0.001. (B) Caspase-11 expression, IL-1β maturation and inflammasome priming, analysed by western blot of cell lysates and supernatants from wild-type BMDM stimulated with attenuated, gamma-irradiated *C*. *trachomatis* particles (γ-CT) or infection with live *C*. *trachomatis* (CT) for 24hrs. (C) Cell death analysed by LDH release in wild-type BMDM in response to stimulation with attenuated, gamma-irradiated *C*. *trachomatis* particles (γ-CT) or infection with live *C*. *trachomatis* (CT) for 24hrs. Data represent the mean from BMDM obtained from three-individual mice, error bars indicate ±SEM. **p = <0.01, ***p = <0.001 ns indicates no significant statistical difference.

We next investigated whether inflammasome responses to *C*. *trachomatis* infection required factors produced by the live-replicating organism. We attenuated *C*. *trachomatis* using gamma-irradiation or used a Chlamydia Protease-like Activity Factor (CPAF) deficient mutant strain that exhibited delayed growth kinetics in Hela cells [28]. Attenuated *C*. *trachomatis* (both gamma-irradiated and the CPAF mutant) failed to induce IL-1β secretion and cell death (Fig 3A and 3C and S2 Fig) but still induced pro IL-1β expression indicating that the attenuated *C*. *trachomatis* particles were not biologically inert and pathogen recognition receptor (PRR) and NFκB responses were intact, but an inflammasome activatory signal was absent (Fig 3B). Importantly, we confirmed that lack of inflammasome activation was not due to reduced internalisation of attenuated *C*. *trachomatis* particles as intracellular staining demonstrated equivalent levels to live *C*. *trachomatis* (S3 Fig) to the attenuated forms. We also noted that although pro IL-1β priming was normal in response to attenuated *C*. *trachomatis*, expression of caspase-11 was markedly reduced in cells stimulated with irradiated but not CPAF deficient *C*. *trachomatis*. This indicated that replication or metabolism of *C*. *trachomatis* intracellularly, provided additional, essential signals required for inflammasome activation and caspase-11 expression in addition to CPAF function.

### NADPH oxidase regulates *C*. *trachomatis* replication within BMDM and human mDC

Our experiments utilising attenuated *C*. *trachomatis* indicated that intracellular replication could be contributing to signals that activate the inflammasome. We therefore wished to identify macrophages that were permissive to *C*. *trachomatis* replication to investigate this hypothesis further.

The NADPH oxidase system is a key component of host defence against microbial pathogens via the generation of anti-microbial reactive oxygen species (ROS). Individuals with inherited, disabling mutations of genes that contribute to the NADPH oxidase system, develop a primary immunodeficiency termed chronic granulomatous disease (CGD) that is characterised by recurrent bacterial and fungal infections [29]. Generation of ROS by NADPH oxidase contributes to the anti-microbial action of macrophages in response to infection with bacteria such as Salmonella [30]. Previous studies using chemical inhibition of NADPH oxidase in Hela cells suggested that ROS amplify *C*. *trachomatis* replication through a caspase-1 mediated mechanism [31]. However, the role of NADPH oxidase in the control of *C*. *trachomatis* replication within primary macrophages is unknown.

We therefore investigated *Chlamydia trachomatis* growth in murine macrophages or human monocyte derived dendritic cells obtained from CGD patients deficient in gp91 Phox (*Cybb)*; a critical component of the NADPH oxidase system in phagocytic cells.

**Fig 4:**
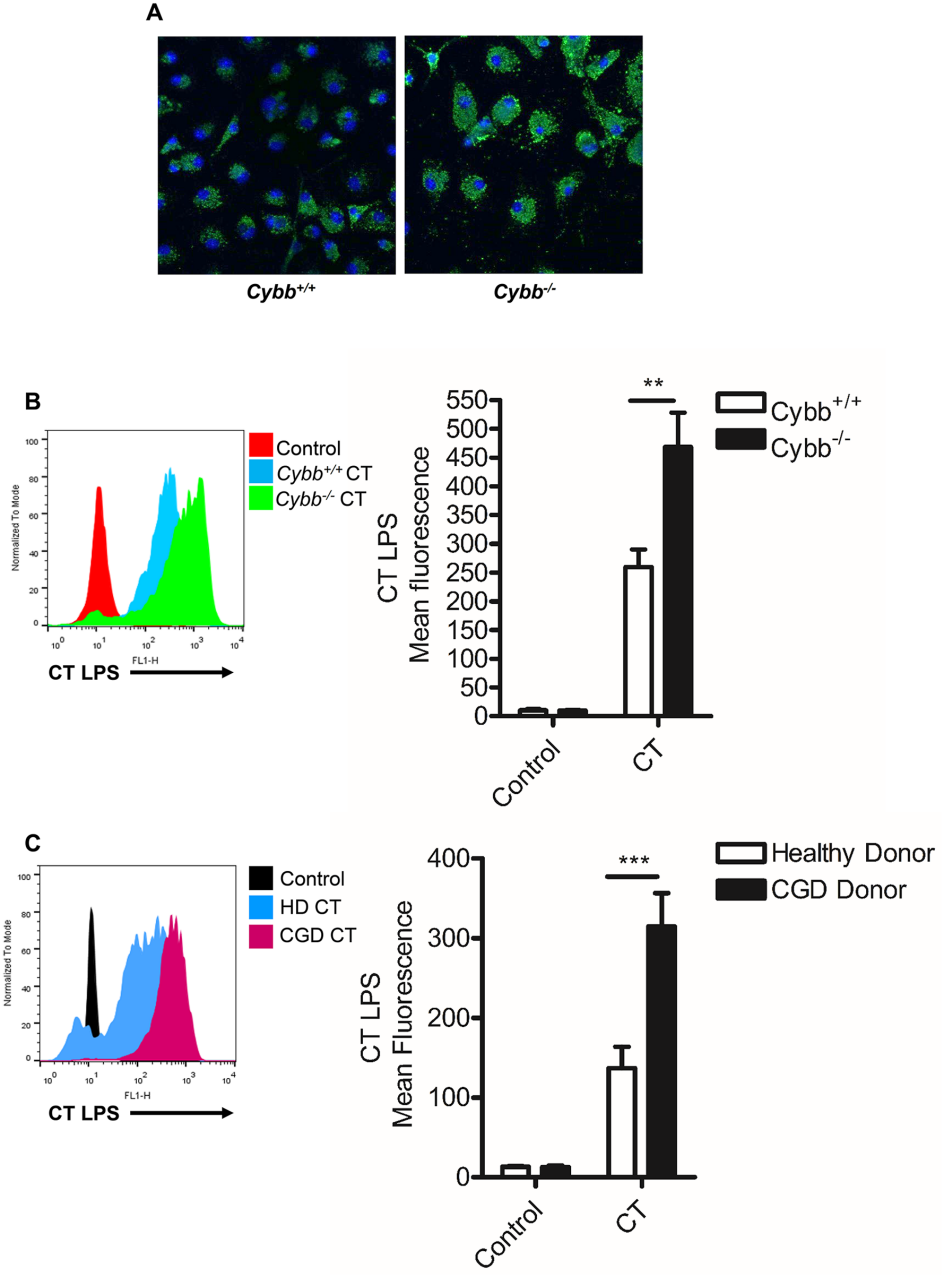
NADPH oxidase controls *C*. *trachomatis* intracellular replication in BMDM and mDC. (A) Assessment of intracellular *C*. *trachomatis* burden at 24hrs post-infection in wild-type (*Cybb*^*+/+*^) and NADPH oxidase deficient (*Cybb*^*-/-*^) BMDM by confocal fluorescent microscopy of *Chlamydia* LPS (green) and nuclei counter-stain with DAPI (blue). (B) Representative FACS histogram plot of *C*. *trachomatis* LPS staining 24hrs post-infection in wild-type (*Cybb*^*+/+*^) or NADPH oxidase deficient (*Cybb*^*-/-*^) BMDM (left panel) and Quantification of geo-mean fluorescence of *Chlamydia* LPS staining in wild-type (*Cybb*^*+/+*^) or NADPH oxidase deficient (*Cybb*^*-/-*^) BMDM infected with *C*. *trachomatis* for 24hrs (right panel). Data represent the mean from BMDM obtained from three-individual mice, error bars indicate ±SEM **p = <0.01. (C) Representative FACS histogram plot of *C*. *trachomatis* LPS staining 24hrs post-infection in healthy-donor or NADPH oxidase deficient CGD patient mDC (left panel) and quantification of geo-mean fluorescence of *Chlamydia* LPS staining in healthy or CGD mDC infected with *C*. *trachomatis* for 24hrs (right panel). Data represent the mean from mDC obtained from four-individual donors from each group, error bars indicate ±SEM, ***p = <0.001.

Intracellular staining of *C*. *trachomatis* LPS and analysis of Chlamydia 16s RNA expression was employed to determine the bacterial burden within infected cells Significantly, infection of macrophages deficient in gp91 phox exhibited a marked increase in the burden of *C*. *trachomatis* compared to wild-type controls (Fig 4A). The intracellular staining of *Chlamydia* LPS in murine macrophages (Fig 4B) or human dendritic cells (Fig 4C) was also quantified using FACS and further confirmed by analysis of *Chlamydia* 16s RNA in BMDM (S4 Fig), a technique employed by others to analyse *Chlamydia* replication [31]. Both murine and human cells deficient in gp91 phox displayed a significant increase in intracellular *C*. *trachomatis* burden compared to control (gp91 phox sufficient) cells, indicating that NADPH oxidase activity was a key regulator of *C*. *trachomatis* replication and survival within myeloid cells in both mice and humans.

### NADPH oxidase deficient cells exhibit elevated inflammasome activation in response to live *C*. *trachomatis* infection but not sterile agonists

**Fig 5:**
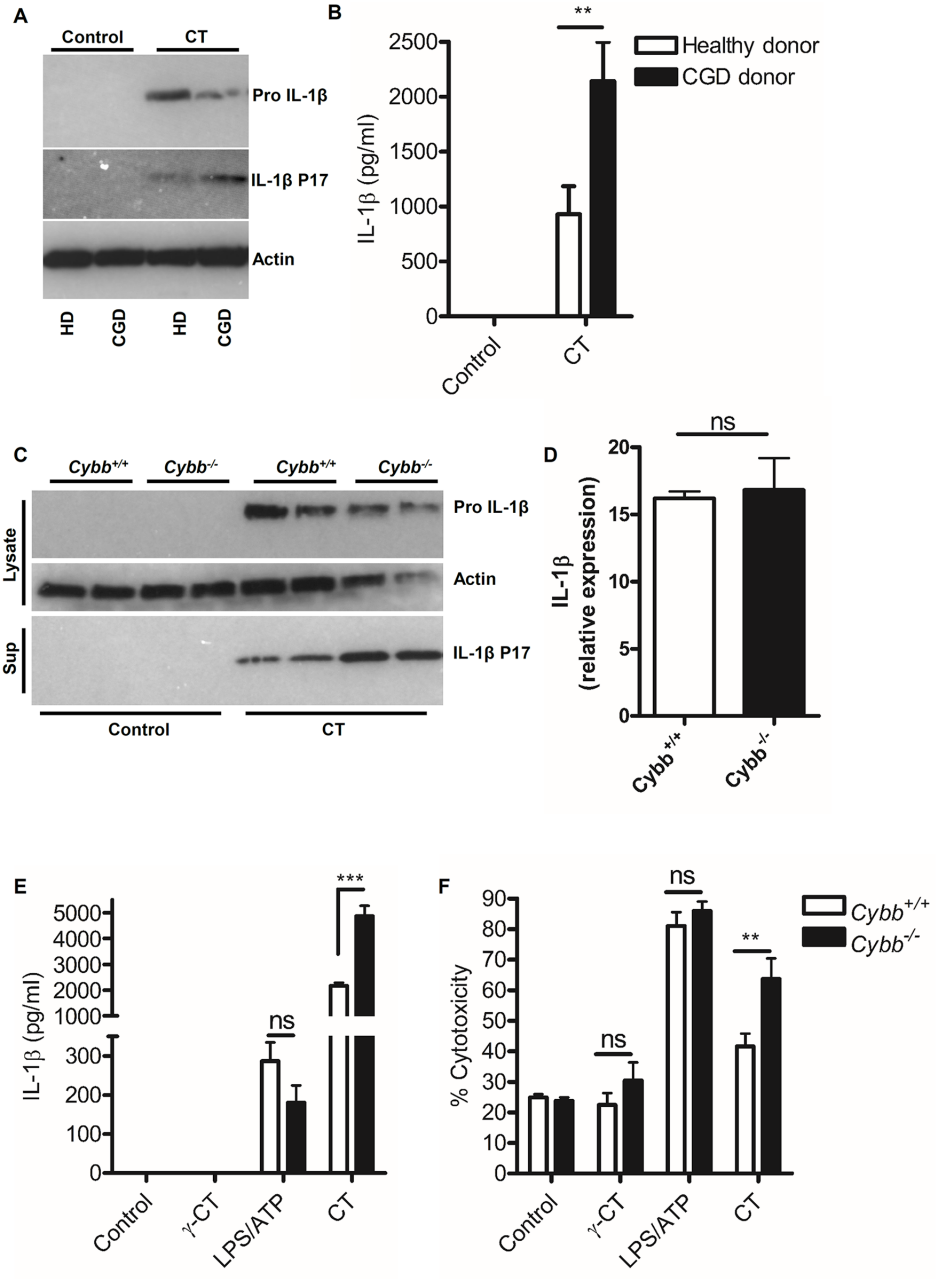
Increased *C*. *trachomatis* replication is associated with elevated inflammasome activation. (A) IL-1β maturation in response to *C*. *trachomatis* infection, analysed by western blot of cell lysates in mDC obtained from healthy donors (HD) or CGD donors (CGD). Data representative of three-independent donors in each patient group. (B) IL-1β secretion analysed by ELISA from mDC obtained from healthy donors or CGD patient donors infected with *C*. *trachomatis* (CT) for 24hrs. Data represents the mean from mDC obtained from seven-donors from each group, error bars indicate ±SEM **p = <0.01. (C) IL-1β maturation in response to *C*. *trachomatis* infection, analysed by western blot of cell lysates and supernatants from wild-type (*Cybb*^*+/+*^) or NADPH oxidase deficient (*Cybb*^*-/-*^) BMDM. Data represents the response from BMDM obtained from two-individual mice. (D) IL-1β mRNA expression relative to HPRT analysed by qRT-PCR of RNA obtained from wild-type (*Cybb*^*+/+*^) or NADPH oxidase deficient (*Cybb*^*-/-*^) BMDM following infection with *C*. *trachomatis* for 6-hours. Data represent the mean from three-individual mice, error bars indicate ±SEM, ns indicates no significant difference. (E) IL-1β secretion analysed by ELISA or (F) Cell death analysed by LDH release from BMDM obtained from wild-type (*Cybb*^*+/+*^) or NADPH oxidase deficient (*Cybb*^*-/-*^) mice following stimulation with gamma-attenuated *C*. *trachomatis* (γ-CT), live *C*. *trachomatis* (CT) or LPS/ATP for 24hrs. Data represent the mean from 12-individual mice (CT) or six-individual mice (control, γ-CT and LPS/ATP), error bars indicate ±SEM. **p = <0.01, ***p = <0.001, ns indicates no significant difference.

Given that we had observed an apparent requirement for *C*. *trachomatis* replication or metabolism in the induction of inflammasome activation, we tested the hypothesis that increased *C*. *trachomatis* replication observed in NADPH oxidase deficient cells, would correspond with increased inflammasome activation. *C*. *trachomatis* infection of dendritic cells from CGD patients (Fig 5A and 5B) or gp91 phox deficient murine macrophages (*Cybb*^-/-^*)* (Fig 5C, 5E and 5F) resulted in increased IL-1β maturation and pyroptosis indicating increased inflammasome activation. Furthermore, expression of IL-1β mRNA was equivalent in wild-type and gp91 phox deficient cells in response to infection indicating that the increased bacterial burden was having a direct effect on inflammasome activation, and was not just due to increased priming (Fig 5D). Crucially, inflammasome responses to sterile agonists such as LPS/ATP or gamma attenuated *C*. *trachomatis*, were not elevated in the absence of NADPH oxidase activity, indicating that the increased bacterial burden was the critical factor leading to inflammasome activation and that this was not simply a consequence of elevated background inflammasome activation in the absence of NADPH oxidase. These data also reinforced our hypothesis that aspects of *C*. *trachomatis* metabolism or replication were factors influencing inflammasome activation.

### *Chlamydia trachomatis* replication and metabolism contribute to type-1 interferon responses in macrophages

**Fig 6:**
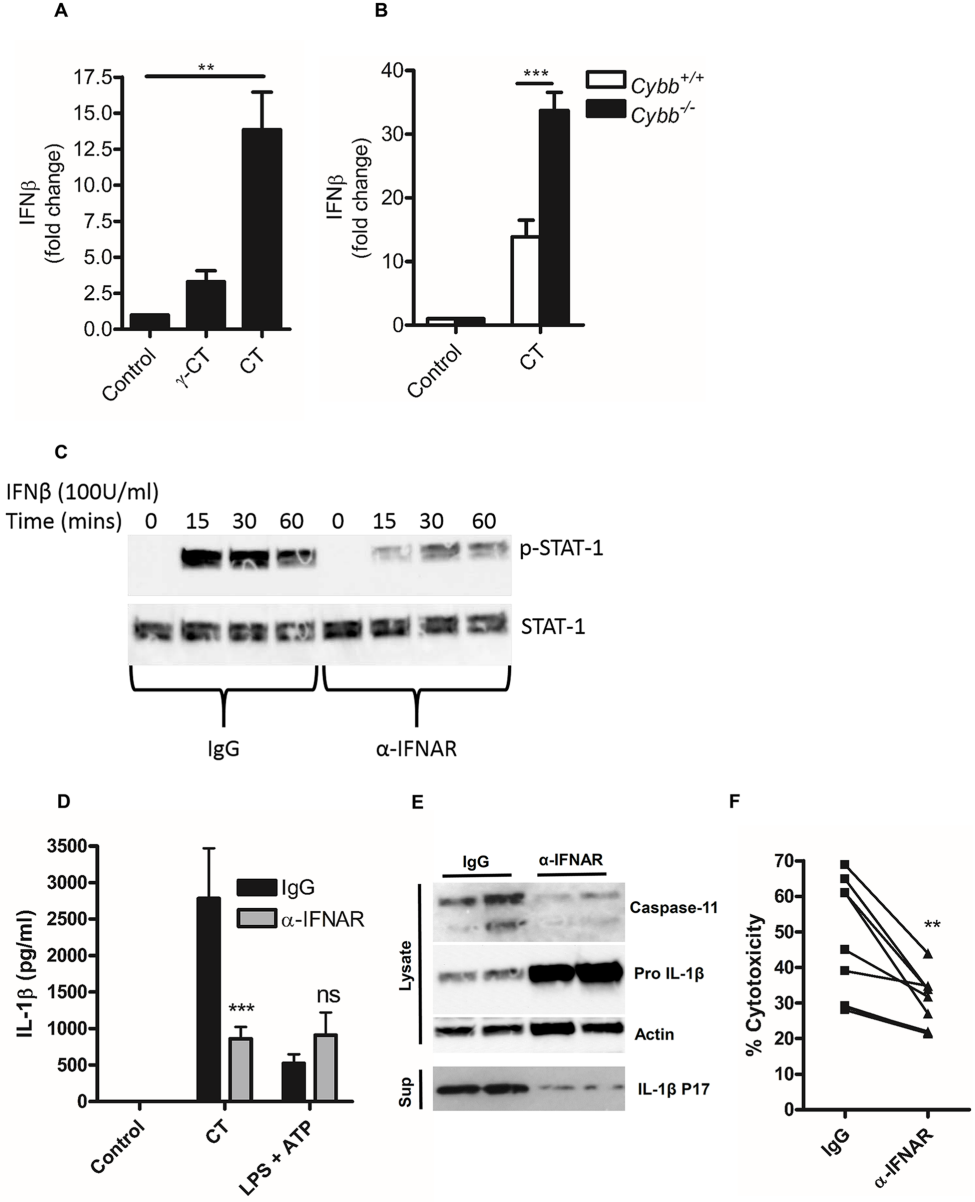
Type-1 interferon signalling regulates *C*. *trachomatis* inflammasome activation. (A) Induction of IFNβ mRNA expression in wild-type BMDM analysed by quantitative RT-PCR stimulated with gamma attenuated *C*. *trachomatis* (γ-CT) or live *C*. *trachomatis* (CT) for 8hrs. (B) Induction of IFNβ mRNA expression in wild-type BMDM (*Cybb*^*+/+*^) or NADPH oxidase deficient BMDM (*Cybb*^*-/-*^) in response to infection with live *C*. *trachomatis* for 8hrs. Data represent the mean from BMDM obtained from three-individual mice in each group, error bars indicate SEM **p = <0.01, ***p = <0.001. (C) STAT-1 phosphorylation analysed by western blot from BMDM stimulated with 100U/ml IFNβ for indicated times in the presence of isotype (IgG) or IFNAR blocking antibody (α-IFNAR). (D) IL-1β secretion analysed by ELISA of supernatants from wild-type BMDM infected with *C*. *trachomatis* (CT) or stimulated with LPS/ATP in the presence of an IFNAR blocking antibody (αIFNAR) or isotype control (isotype IgG) for 24hrs. Data represent the mean from BMDM obtained from nine-individual mice, error bars indicate ±SEM ***p = <0.001. (E) Caspase-11 expression and IL-1β maturation analysed by western blot of lysates and supernatants from wild-type BMDM in response to *C*. *trachomatis* infection in the presence of an IFNAR blocking antibody (αIFNAR) or isotype control (isotype IgG) for 24hrs. (F) Cell death analysed by LDH release from wild-type BMDM in response to *C*. *trachomatis* infection (CT) in the presence of an IFNAR blocking antibody (αIFNAR) or isotype control (isotype IgG) for 24hrs. Data represent the mean from BMDM obtained from nine-individual mice, error bars indicate ±SEM **p = <0.01.

Recently, type-1 interferon signalling has been demonstrated to play an important role in host defence and inflammasome activation to bacterial pathogens [32] and is crucial for the expression of caspase-11 in response to a range of gram-negative bacteria [17]. We therefore investigated whether intracellular metabolism or replication of *C*. *trachomatis* within macrophages contributed to type-1 interferon expression. Significantly, gamma-irradiated attenuated *C*. *trachomatis* or the CPAF deficient mutant (S5 Fig) failed to induce significant interferon-β expression compared to non-attenuated bacteria (Fig 6A). Furthermore, gp91 phox deficient macrophages, harbouring increased bacterial burdens also displayed elevated interferon-β expression compared to wild-type controls (Fig 6B). These data indicated that type-1 interferon responses could be critical mediators of inflammasome activation in macrophages during *C*. *trachomatis* infection. We therefore tested the hypothesis that autocrine, type-1 interferon signalling through the type-1 interferon receptor, IFNAR, contributed to inflammasome activation during *C*. *trachomatis* infection. To do this, we infected macrophages with *C*. *trachomatis* in the presence of an IFNAR blocking antibody or isotype control. We confirmed the effectiveness of blocking IFNAR by examining STAT-1 phosphorylation in response to interferon-β stimulation of wild-type BMDM (Fig 6C). Blocking IFNAR during *C*. *trachomatis* infection resulted in reduced IL-1β secretion (Fig 6D), IL-1β maturation, caspase-11 expression (Fig 6E) and cell death (Fig 6F) but, as expected, did not significantly affect canonical inflammasome activation with LPS/ATP. Furthermore, the effect on IL-1β secretion was of reduced proteolytic processing and not priming of pro IL-1β, as blocking IFNAR during *C*. *trachomatis* infection actually resulted in increased pro IL-1β, presumably due to reduced maturation by caspase-1 and accumulation of the un-processed substrate and the inhibitory effect of interferon signalling on pro IL-1β synthesis [16] (Fig 6E). These data suggested that *C*. *trachomatis* replication or metabolism induced a type-1 interferon signature that controlled both canonical and non-canonical inflammasome activation.

### STING regulates type-1 interferon expression and inflammasome activation in response to *C*. *trachomatis* infection

**Fig 7:**
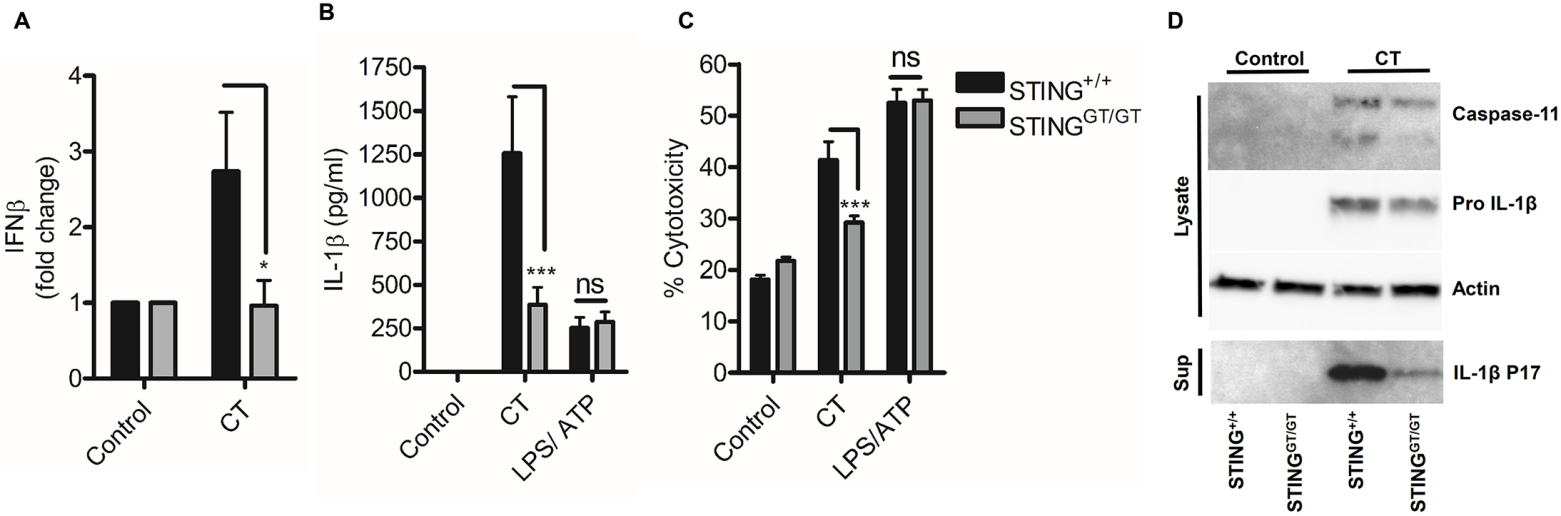
STING regulates interferon induced inflammasome activation in response to *C*. *trachomatis* infection. (A) Induction of IFNβ mRNA in wild-type (STING^+/+^) or STING deficient (STING^GT/GT^) BMDM infected with *C*. *trachomatis* (CT) for 8hrs. Data represent the mean of BMDM obtained from six-individual mice, error bars indicate SEM *p = <0.05. (B) IL-1β secretion analysed by ELISA of supernatants from wild-type (STING^+/+^) or STING deficient (STING^GT/GT^) BMDM infected with *C*. *trachomatis* (CT) or stimulated with LPS /ATP for 24hrs. Data represent the mean of BMDM obtained from twelve-(CT) or six-(LPS/ATP) individual mice, error bars indicate SEM *p = <0.05, **p = <0.01, ***p = <0.001. (C) Cell death analysed by LDH release in wild-type (STING^+/+^) or STING deficient (STING^GT/GT^) BMDM. Data represent the mean from BMDM obtained from six-(CT) or three-(LPS/ATP) individual mice, error bars indicate SEM *p = <0.05, **p = <0.01, ***p = <0.001. (D) Capase-11 expression and IL-1β maturation analysed by western blot of lysates and supernatants from wild-type (STING^+/+^) or STING deficient (STING^GT/GT^) BMDM infected with *C*. *trachomatis*.

Stimulator of interferon gene (STING) is a critical mediator of type-1 interferon expression in response to *Chlamydia sp* infection [24, 33, 34]. Given that we have demonstrated a crucial role for autocrine type-1 interferon signalling in *C*. *trachomatis* induced inflammasome activation, we tested the hypothesis that STING was a central regulator of inflammasome activation. Infection of macrophages from STING deficient mice with *C*. *trachomatis* failed to induce interferon-β expression (Fig 7A) and exhibited reduced IL-1β secretion (Fig 7B), cell death (Fig 7C) and reduced IL-1β processing and caspase-11 expression (Fig 7D) but had equivalent responses to LPS/ATP stimulation. Thus, STING is a critical mediator of interferon dependent inflammasome activation in response to *C*. *trachomatis* infection of macrophages.

### STING dependent canonical inflammasome responses to *C*. *trachomatis* infection are independent of cGAS

**Fig 8:**
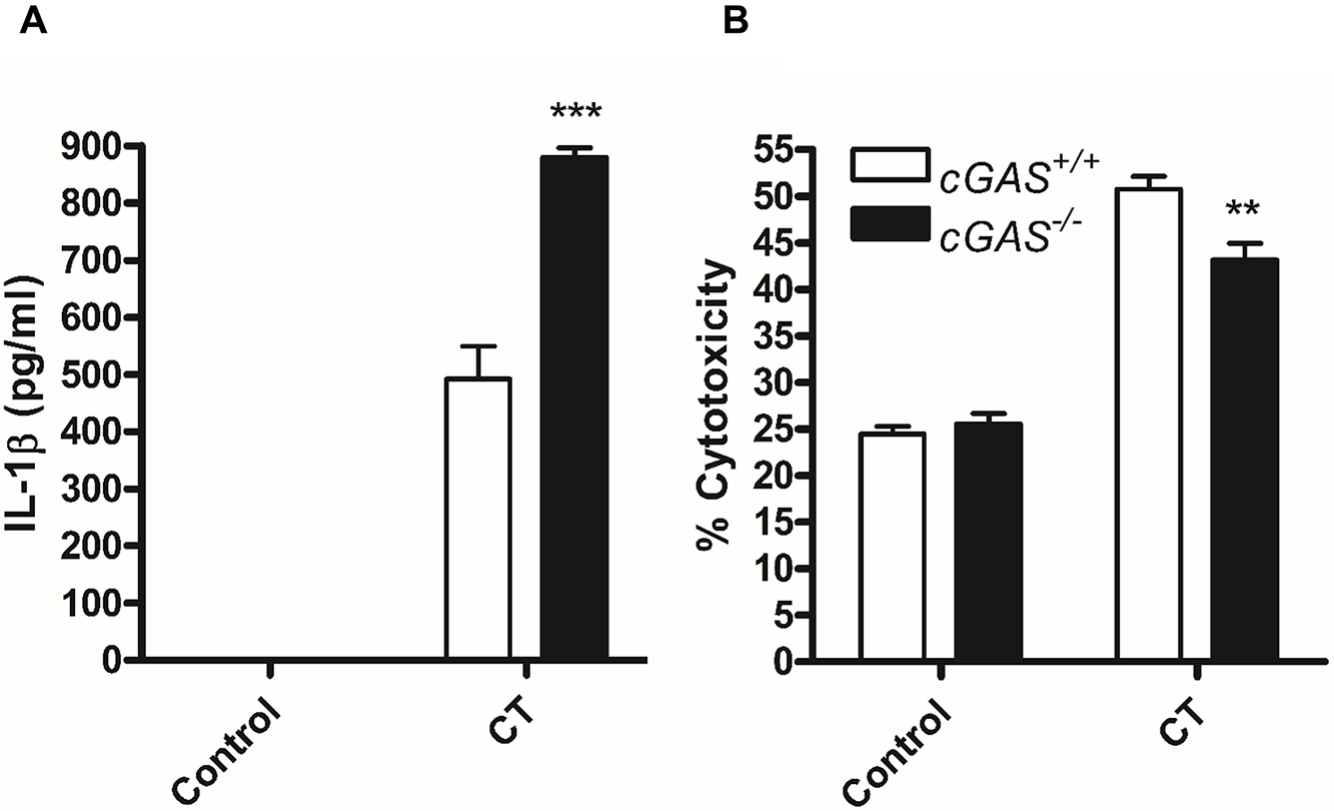
cGAS is not required for canonical inflammasome responses to *C*. *trachomatis* infection. (A) IL-1β release measured by ELISA or (B) cell death measured by LDH release in supernatants from wild-type (cGAS^+/+^) or cGAS deficient (cGAS^-/-^) BMDM infected with *C*. *trachomatis* (CT) for 24hrs. Data represent the mean from BMDM obtained from three individual mice, error bars indicate ±SEM **p = <0.01, ***p = <0.001.

Activation of STING to induce type-1 interferon responses can occur through two distinct pathways: 1. the conversion of cytosolic DNA to cGAMP catalysed by cGAS which is then recognised by STING or 2. the direct recognition of cyclic di-nucleotides (cyclic di-AMP/GMP) produced by certain bacteria. Recently, cGAS was shown to be crucial for STING dependent inflammasome responses to *Francisella tularensis* infection of macrophages [35, 36]. We therefore analysed whether cGAS was required for STING mediated inflammasome activation in response to *C*. *trachomatis* infection using cGAS deficient BMDM. In contrast to inflammasome responses to *F*. *tularensis*, cGAS deficient BMDM produced elevated IL-1β in response to *C*. *trachomatis* infection indicating that STING dependent canonical caspase-1 activation through NLRP3 and AIM2 activation was independent of cGAS conversion of host or microbial DNA to cGAMP (Fig 8A). Surprisingly however, there was a small but significant protection from *C*. *trachomatis* induced pyroptosis in cGAS deficient cells (Fig 8B). Given that we demonstrate cell death responses are delayed compared to IL-1β release and death is governed by both canonical activation of caspase-1 and non-canonical caspase-11 activation, suggests that later release of microbial DNA to the cytosol may amplify STING responses that are not required for canonical inflammasome activation.

### The bacterial metabolite cyclic di-AMP recovers STING dependent inflammasome responses to attenuated *C*. *trachomatis*

**Fig 9:**
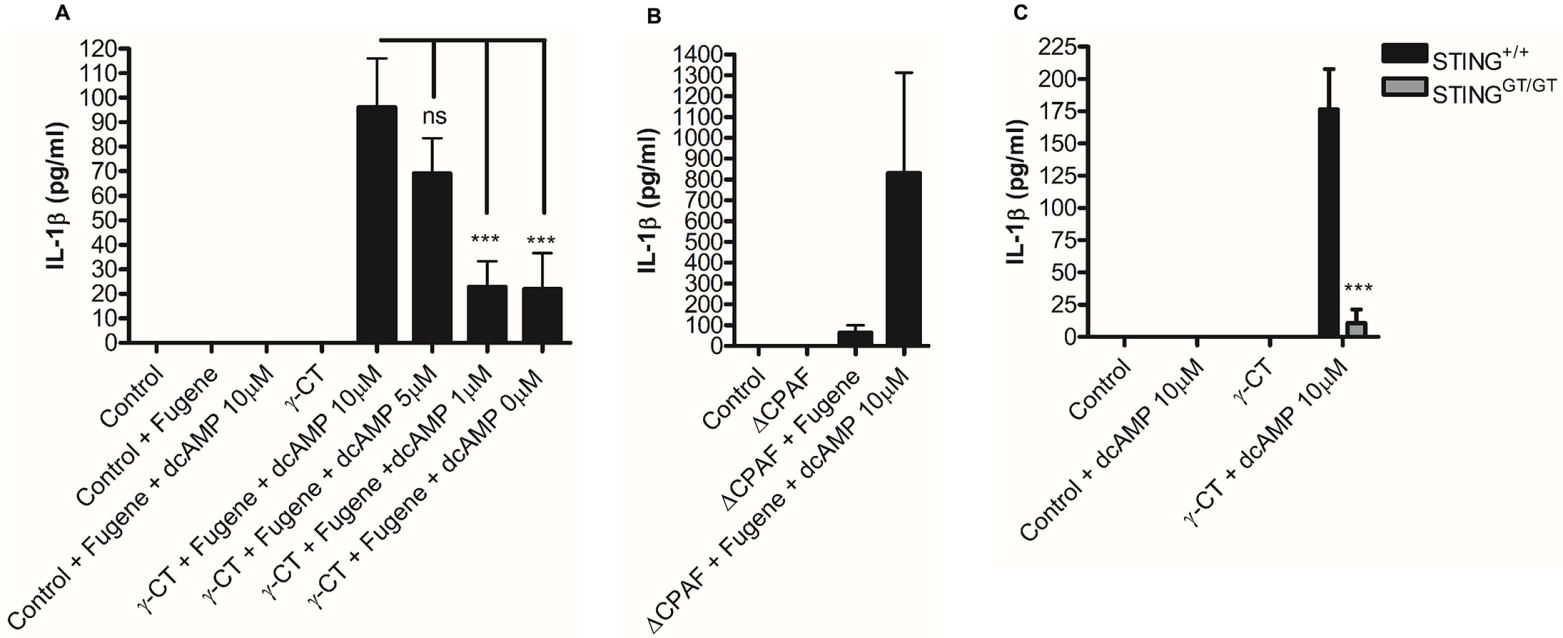
Exogenous cyclic di-AMP recovers inflammasome activation in response to attenuated *C*. *trachomatis*. (A) IL-1β secretion analysed by ELISA in wild type BMDM in response to stimulation with gamma attenuated *C*. *trachomatis* (γ-CT) transfected with cyclic di-AMP (dcAMP) at indicated concentrations. Data represent the mean of BMDM obtained from six to nine individual mice, error bars indicate ±SEM. (B) IL-1β secretion analysed by ELISA of supernatants from wild-type BMDM following infection with CPAF deficient *C*. *trachomatis* (ΔCPAF) transfected with 10μM cyclic di-AMP (dcAMP) for 24hrs. Data represented as the mean of one experiment performed on cells from three individual mice, error bars indicate ±SEM. (C) IL-1β secretion analysed by ELISA in wild-type (STING^+/+^) or STING deficient (STING^GT/GT^) in response to stimulation with gamma attenuated *C*. *trachomatis* (γ-CT) transfected with cyclic di-AMP (dcAMP) (10μM). Data represent the mean of BMDM from three-individual mice, error bars indicate ±SEM *p = <0.05, **p = <0.01, ***p = <0.001.

STING induced interferon responses during *C*. *trachomatis* infection, have been demonstrated to occur as a consequence of recognition of a metabolite; cyclic di-AMP, that is produced by metabolically active *C*. *trachomatis* [33]. Given that we identified STING as a critical regulator of canonical and non-canonical inflammasome responses to *C*. *trachomatis* infection and that cGAS was not required for canonical caspase-1 activation to induce IL-1β release in addition to finding a requirement for *C*. *trachomatis* metabolism or replication in the induction of interferon-β expression and subsequent inflammasome activation, we investigated whether cyclic di-AMP was a critical factor in STING mediated activation of the inflammasome. To test this hypothesis, we stimulated wild-type BMDM with attenuated *C*. *trachomatis* that failed to induce inflammasome activation and transfected the metabolite cyclic di-AMP in to the cells. Crucially, transfection of titrated amounts of cyclic di-AMP in to cells stimulated with attenuated gamma irradiated (Fig 9A) or CPAF deficient (Fig 9B) *C*. *trachomatis* resulted in partial recovery of IL-1β secretion that was dependent on STING (Fig 9C). These data provide an attractive explanation as to how STING mediated inflammasome responses could be regulated in the absence of a requirement for cGAS conversion of DNA to cGAMP during infection with *C*. *trachomatis* that requires a replicating or metabolically active organism.

## Discussion

Innate host defence relies upon a repertoire of pathogen recognition systems that detect a diverse array of conserved microbial components in order to initiate inflammatory responses. Here, we provide evidence that inflammasome activation in response to *Chlamydia trachomatis* infection is dependent on detection of a microbial metabolite produced by the live organism. The inflammasome is an important component of innate host defence against bacterial pathogens including *Chlamydia sp*, but excessive or inappropriate activation contributes to infection associated immunopathology. Therefore, inflammasome activation must be tightly regulated. Our data demonstrate a novel mechanism of inflammasome regulation that allows the host to differentiate between live (a potential infectious threat) or attenuated *Chlamydia trachomatis* and mount appropriate responses. We suggest that this distinction is critical for effective host defence, but also regulates inappropriate or excessive responses by only responding to live organisms. Conversely, these data also suggest a potential mechanism of immune evasion that could be adopted by *C*. *trachomatis* by modulating production of cyclic di-AMP. Bacterial pathogens are known to evade non-canonical inflammasome activation by modulating the acylation status of their lipid-A moieties [9], and we suggest that modulating production of inflammasome activating metabolites could also be a strategy utilised by pathogenic bacteria to evade this response.

We have shown that STING, independent of cGAS, is a critical component of interferon mediated inflammasome activation in response to *C*. *trachomatis* infection, and differs from recent studies utilising *F*. *tularensis* that required conversion of bacterial DNA to cGAMP, rather than detection of a microbial metabolite to induce STING mediated AIM2 inflammasome activation [35, 36]. These previous studies raise an important paradoxical question because AIM2 activation also requires the release of DNA from the pathogen in an interferon dependent process, yet the interferon signal also requires conversion of this same DNA by cGAS to induce the STING mediated interferon signal. This would suggest that either cGAS is a more sensitive sensor of DNA than AIM2 and responds to low levels within the cytosol that would not initiate AIM2 activation or that something else is providing the initiating interferon signal leading to loss of compartmentilisation and amplification of responses. We envisage that in our model—microbial metabolism and the production of cyclic di-AMP precedes any involvement of cGAS that is only partially required for cell death responses. Instead, cyclic di-AMP induction of STING activation results in small, but significant increases in type-1 interferon expression that facilitates the up-regulation of IRG’s and GBP’s that are known to be recruited to Chlamydial containing vacuoles [37–39]. Damage to the *Chlamydia* containing vacuole, mediated by IRG’s and GBP’s, could then result in the further release of vacuole contents including microbial DNA that further enhances STING activation through cGAS [34] and also activates the AIM2 inflammasome as we and others have reported recently [23]. In this regard, we would not discount cGAS from *C*. *trachomatis* induced inflammasome activation, but would suggest it enhances cell death responses, rather than initiates, the response. Furthermore, our experiments using attenuated *C*. *trachomatis* support this hypothesis, as attenuated organisms did not activate the inflammasome despite containing a full complement of unaltered DNA and LPS that would be immunostimulatory if detected by cytosolic receptors. This suggests that a loss of compartmentalisation of the *Chlamydia* containing vacuole is a key event that delivers inflammasome activatory ligands to the cytosol. Thus release of cyclic di-AMP activates STING which in turn mediates type-1 interferon signalling.

We also provide evidence that cell death observed during *C*. *trachomatis* is true pyroptosis. Deletion of both caspase-1 and caspase-11 prevented cell death and this observation would rule out other forms of lytic cell death such as necroptosis. It is unusual that both caspases could induce pyroptosis, but this is not without precedent [40], and has also been reported for *Chlamydia* infection by others independently of our studies [23]. We think it is also important that cell death was delayed compared to IL-1β release and suggests IL-1β release is controlled via an active process rather than accidental release as a consequence of cell death, although we cannot rule out that sensitivity of the assays employed could also contribute to these findings. However, in support of our findings, Recent studies have identified that cleavage of GSDMD by inflammatory caspases is a critical mediator of pyroptosis [11, 12, 41] and IL-1β release [12, 41]. It is also hypothesised that the number of gasdermin pores formed with the plasma membrane of the cell could control the balance between release of mature cytokine and commitment to cell death so that fewer pores allow release of IL-1β without cell death [42]. This would be an attractive explanation of the asynchronous behaviour of IL-1β release and cell death responses observed during *C*. *trachomatis* infection. Given that *C*. *trachomatis* induced pyroptosis could utilise both caspases, the role of GSDMD would be an interesting line of enquiry and will form part of our future studies. Furthermore, although inflammatory caspases are known to play a role in *Chlamydia* induced immunopathology [3], the role of pyroptosis has yet to be defined.

Finally, we have demonstrated a striking role for the control of intracellular growth of *C*. *trachomatis* by the NADPH oxidase system in both human and murine cells. Increased intracellular growth correlated with elevated inflammasome activation reinforcing our hypothesis that *C*. *trachomatis* replication or metabolism is a critical factor inducing inflammasome activation. There are very few published data investigating the role of NADPH oxidase during *Chlamydia* infection. Previous studies have utilised chemical inhibition to study ROS function during *Chlamydia* infection of Hela cells which suggested that ROS were important for *C*. *trachomatis* replication within these cells [31]. However, Hela cells provide a wholly different cellular environment to primary macrophages and *C*. *trachomatis* exists within a specially constructed pathogen containing vacuole that may well protect the organism from the toxic effects of ROS generation within non-phagocytic cells. It is not well defined which compartment *C*. *trachomatis* occupies within macrophages. We speculate from our work that this compartment is likely to be formed as a consequence of phagocytic activity as uptake of the bacteria was independent of high speed centrifugation often employed to achieve infection of non-phagocytic cells such as Hela. We would therefore suggest that *Chlamydia trachomatis* is likely to be existing within a different compartment in macrophages compared to Hela cells and therefore may be more susceptible to the toxic effects of ROS generated through NADPH oxidase activity which could account for the intriguing differences between our findings and others. Intriguingly, other members of the *Chlamydiaceae* family have been shown to express functional catalase activity [43] suggesting that ROS is an important host defence mechanism against *Chlamydia* infection and may limit the replicative ability of *C*. *trachomatis* within phagocytic cells such as macrophages. Importantly, a recent report has linked macrophage pyroptosis and neutrophil oxidative killing of bacteria as an important axis in innate host defence against bacterial infection [44]. Neutrophils from CGD patients display reduced bactericidal effects on *Chlamydia trachomatis* [45]. We would therefore predict that CGD patients may be at risk from more severe immunopathology during *C*. *trachomatis* infection due to elevated inflammasome responses of macrophages and reduced bacterial killing by neutrophils. This would also be in agreement with the consensus that CGD patients have a pro-inflammatory phenotype [46].

In summary, we propose that intracellular replication of *Chlamydia trachomatis* and production of the metabolite cyclic di-AMP is a key pathogen associated molecular pattern detected by STING that is crucial for activation of both canonical and non-canonical inflammasomes. This requires type-1 interferon and allows the host to initiate appropriate immune responses. We also suggest that modulation of cyclic di-AMP production by *Chlamydia trachomatis* could provide a mechanism of immune evasion and contribute to mechanisms of infection latency. Finally, targeting of the STING/interferon pathway may provide useful vaccine adjuvant and therapeutic targets to aid the treatment of *Chlamydia trachomatis* infection and its associated inflammatory pathology.

## Materials and methods

### Ethics statement

All studies involving human subjects were performed in accordance with the Declaration of Helsinki, with approval of the Cambridge Regional Ethical Committee (01/363). All donors gave written informed consent.

All animal related work was conducted by trained and appropriately licensed staff, under the authority and conditions of a Home Office project licence. This licence is issued under the UK Animal (Scientific Procedures) Act 1986 (ASPA), following local ethical approval. Home Office Inspectors provide governmental supervision of the work. Local ethical approval and supervision of standards of work and animal husbandry is carried out by the University of Cambridge Animal Welfare Ethical Review Body (AWERB) and its delegated representatives. Animals used for tissue were culled by fully trained personnel using approved humane methods under Schedule 1 of ASPA.

### Cell culture and infection

Bone marrow derived macrophages (BMDM) were prepared from femurs of littermate wild type and genetically deficient animals by culturing bone marrow isolates for 7-days in RPMI containing 10% v/v HIFCS, L-glutamine, 5% v/v L929 conditioned medium with gentamicin. Of note, we became aware that macrophages produced from mice housed in different facilities displayed extremely variable responses to stimulation. It was therefore imperative that comparisons between wild-type and knock-out animals were made using littermates or, as a minimum, using age and sex matched mice housed in the same facility, and not between mice housed at different facilities. Unprimed macrophages were infected with live *Chlamydia trachomatis* at a multiplicity of infection (MOI) of 20. Attenuated *Chlamydia trachomatis* was achieved by γ-irradiating live *C*. *trachomatis* elementary bodies purified by high speed density centrifugation for 6-hours in a Gammacell-1000 irradiator (Atomic Energy of Canada Ltd) or using a Chlamydia Protease Activity like Factor (CPAF) deficient *C*. *trachomatis* mutant (ΔCPAF) described previously [28]. Attenuated *C*. *trachomatis* were used at a multiplicity of infection of 20 and attenuation was determined by the absence of replication in Hela cells. Intracellular LPS stimulation of macrophages was achieved by priming BMDM with 100ng/ml LPS for 4-hours in L929 conditioned RPMI. After priming, the supernatants were removed and replaced with serum free optiMEM (Gibco) containing 5μg/ml LPS with 5μl/ml Fugene (Promega). Cells were incubated for 24-hours before supernatants were harvested for ELISA and LDH assays. Recovery of inflammasome activation with attenuated *C*. *trachomatis* was achieved by stimulation of BMDM for 4-hours with γ-attenuated *or* CPAF deficient *C*. *trachomatis* mutant (ΔCPAF) in L929 conditioned RPMI as per intracellular LPS stimulation. The supernatant and non-internalised *C*. *trachomatis* were then removed and the media replaced with optiMEM containing cyclic-di-AMP (Invivogen, France) at indicated concentrations with Fugene. BMDM were then incubated for 24hrs before supernatants were harvested for ELISA.

Human monocyte derived dendritic cells (mDC) were obtained by IL-4/GMCSF differentiation [27, 47] of peripheral blood monocytes from age and sex matched chronic granulomatous disease (CGD) patients (recruited at the Royal Free Hospital, London) or healthy donors, (recruited at the University of Cambridge Department of Medicine, Cambridge) conforming to ethical guidelines of each institution.

### Intracellular staining of Chlamydia for fluorescent microscopy and FACS analysis

Assessment of relative amounts of intracellular *C*. *trachomatis* was determined by fluorescent staining of *Chlamydia* LPS. BMDM or mDC were plated on coverslips (microscopy) or without coverslips (FACS) in 24-well plates at 2.5x10^5^ cells/well and infected as described above for 24-hours. Intracellular staining of LPS was achieved by washing cells three times in PBS before fixation and permeabilisation using CellFix (BD Bioscience, USA) and incubating cells at room temperature with 1μg/ml FITC conjugated monoclonal mouse anti-chlamydia LPS (SourceBioscience U.K.). Cells were washed a further three times and mounted on slides using DAPI counterstain for fluorescent microscopy or scraped and assayed by FACS (FacsCalibur, BD Bioscience, USA). Uninfected cells were stained as described and acted as a negative control.

### ELISA and LDH assay

Detection of mature IL-1β in cell culture supernatants was achieved by ELISA following the manufacturer’s instructions (Mouse or human Ready-SET-Go IL-1β ELISA, Ebioscience, USA). Macrophages were seeded in 96-plates at 1x10^5^ cells/well and cultured for 24hrs in the presence of cell stimulations. For assessment of cell death, a lactate dehydrogenase (LDH) release assay was employed (promega U.K.) following the manufacturer’s instructions. The percentage cytotoxicity was calculated from the absorbance of the test well divided by the absorbance of the corresponding 100% lysis control wells.

### SDS PAGE and western blotting

For analysis of protein expression cytosolic protein extracts and cell culture supernatants were assayed by SDS PAGE and western blotting as described previously [27]. Briefly, equal amounts of cytosolic protein, determined by Bradford Assay (Thermo U.K.) was mixed with an appropriate volume of 6x reducing buffer and boiled for 10 minutes before loading on to 4–20% gradient SDS pre-cast gels (BioRad U.K.). Separated protein was then transferred to PVDF membrane (BioRad U.K.) using a Transblot Turbo (BioRad U.K.). Membranes were blocked for 1-hour at RT in 5% w/v milk protein before incubation with appropriate primary antibody overnight at 4°C. Membranes were washed 3 times in TBS-TWEEN (0.05%v/v) before incubating with appropriate secondary HRP conjugated antibodies for 1-hour at RT. Membranes were developed via ECL (Lightning super signal, Perkin Elmer, USA) and visualised using a G-box (Syngene U.K.) or chemiluminescence film (Amersham Hyperfilm GE Healthcare U.K). Membranes were stripped using low pH stripping buffer for 30-minutes at RT followed by blocking and incubation with antibody. The following primary antibodies were used in this study: Monoclonal rat anti-mouse Caspase-11 (clone 17D9) (Sigma U.K.), Goat anti-mouse IL-1β (R&D Systems U.K.), Goat anti-human IL-1β (R&D Systems U.K.) and monoclonal mouse anti-actin (Abcam U.K.).

### RNA extraction and qRT-PCR

Macrophages were cultured at 0.5x10^6^ cells per well of 24-well plate and were infected with an MOI of 20 live *C*. *trachomatis*, γ-irradiated *C*. *trachomatis* or a CPAF deficient *C*. *trachomatis* for 8hrs. Cells were lysed via addition of RNA lysis buffer (Norgen, Canada) directly to the well. RNA was purified using the Norgen RNA kit according to manufacturer’s instructions. For interferon-β and IL-1β expression qRT-PCR was employed using commercial probe/primer sets (LifeTechnologies U.K.) and analysed using the Taqman ‘one-step’ system (LifeTechnologies U.K.). Analysis of *C*. *trachomatis* 16s expression was determined by quantitative PCR of cDNA prepared from total RNA extracted as above.

### Statistical analysis

All numeric data were analysed using Graphpad Prism (USA). Analysis of multiple data groups with single variables was analysed using 1-way ANOVA with Dunnet’s post-test while multiple data groups with two variables was analysed using a 2-way ANOVA with Bonferonni’s post-test. Comparison of data between two-paired data sets was analysed using a paired Student’s t-test. All data are represented as ±SEM of the mean performed on BMDM obtained from at least three individual mice or mDC obtained from at least three individual human donors unless stated otherwise. A p value <0.05 was deemed significant.

## Supporting information

S1 Fig A mitochondrial anti-oxidant inhibits *C*. *trachomatis* induced canonical inflammasome activation that is independent of IRE1. (A) IL-1β secretion analysed by ELISA of supernatants wild-type BMDM infected with *C*. *trachomatis* (CT) presence of the mitochondrial anti-oxidant; MitoQ (1μM) for 24hrs (B) Pro IL-1β expression analysed by western blotting of lysates from unstimulated or C. trachomatis infected BMDM in the presence (+) or absence (-) of 1μM MitoQ for 24hrs. (C) Cell death analysed by LDH release from wild-type BMDM infected with *C*. *trachomatis* (CT) presence of the mitochondrial anti-oxidant; MitoQ (1μM) for 24hrs. (D) IL-1β secretion analysed by ELISA of supernatants from *C*. *trachomatis* (CT) infected wild-type (IRE-1^+/+^) or IRE1 knock-out (IRE-1^-/-^) BMDM for 24hrs. (E) IL-1β secretion analysed by ELISA of supernatants from wild-type BMDM infected with *C*. *trachomatis* (CT) in the presence of the IRE1 inhibitor 4μ8c (30μM) for 24hrs. Data represented as the mean of one experiment performed on cells from three individual mice, error bars indicate ±SEM. *p = <0.05, **p = <0.01 and ***p = <0.001. ns indicates no statistical significance between samples.
(TIF)

S2 Fig *C*. *trachomatis* induced IL-1β release requires CPAF. (A) IL-1β secretion analysed by ELISA of supernatants from wild-type BMDM infected with CPAF deficient (ΔCPAF) or CPAF sufficient control (CPAF WT) *C*. *trachomatis*. (B) IL-1β maturation and priming analysed by western blotting of cell lysates and supernatants from wild-type BMDM infected with CPAF deficient (ΔCPAF) or CPAF sufficient control (CPAF WT) *C*. *trachomatis* for 24hrs. (C) cell death analysed by LDH release from wild-type BMDM infected with CPAF deficient (ΔCPAF) or CPAF sufficient control (CPAF WT) *C*. *trachomatis* for 24hrs. Data represented as the mean of one experiment performed on cells from three individual mice, error bars indicate ±SEM. (D) Caspase-11 expression analysed by western blotting of lysates from BMDM infected with deficient (ΔCPAF) or CPAF sufficient control (CPAF WT) *C*. *trachomatis* for 24hrs.
(TIF)

S3 Fig Uptake of irradiated and CPAF deficient C. trachomatis by BMDM is comparable to non-attenuated organism. (A) Intracellular staining of *C*. *trachomatis* LPS in BMDM analysed by FACS following infection with irradiated *C*. *trachomatis* (γ-CT) or non-attenuated *C*. *trachomatis* (CT). (B) Intracellular staining of *C*. *trachomatis* LPS in BMDM analysed by FACS following infection with CPAF deficient *C*. *trachomatis* (ΔCPAF) or wild-type *C*. *trachomatis* (WT CT).
(TIF)

S4 Fig Analysis of C. trachomatis 16s expression. *C*. *trachomatis* replication in wild type (Cybb^+/+^) or Cybb deficient (Cybb^-/-^) BMDM analysed by qRT-PCR of *C*. *trachomatis* 16s RNA expression following *C*. *trachomatis* infection for 6-hours. Data represented as the mean of one experiment performed on BMDM from three individual mice, error bars indicate ±SEM *p = <0.05.
(TIF)

S5 Fig *C*. *trachomatis* induced Type-1 interferon response requires CPAF. Induction of IFNβ mRNA expression in wild type BMDM analysed by quantitative RT-PCR following infection with CPAF deficient (ΔCPAF) or CPAF sufficient control (CPAF WT) *C*. *trachomatis* for 8hrs. Data represented as the mean of one experiment performed on cells from three individual mice, error bars indicate ±SEM. *p = <0.05, **p = <0.01.
(TIF)
