# Supplementary figures and images for: Detection of a microbial metabolite by STING regulates inflammasome activation in response to Chlamydia trachomatis infection
Source: PLoS Pathog. 2017 Jun 1;13(6):e1006383. doi: 10.1371/journal.ppat.1006383 (PMC5453623; doi:10.1371/journal.ppat.1006383)

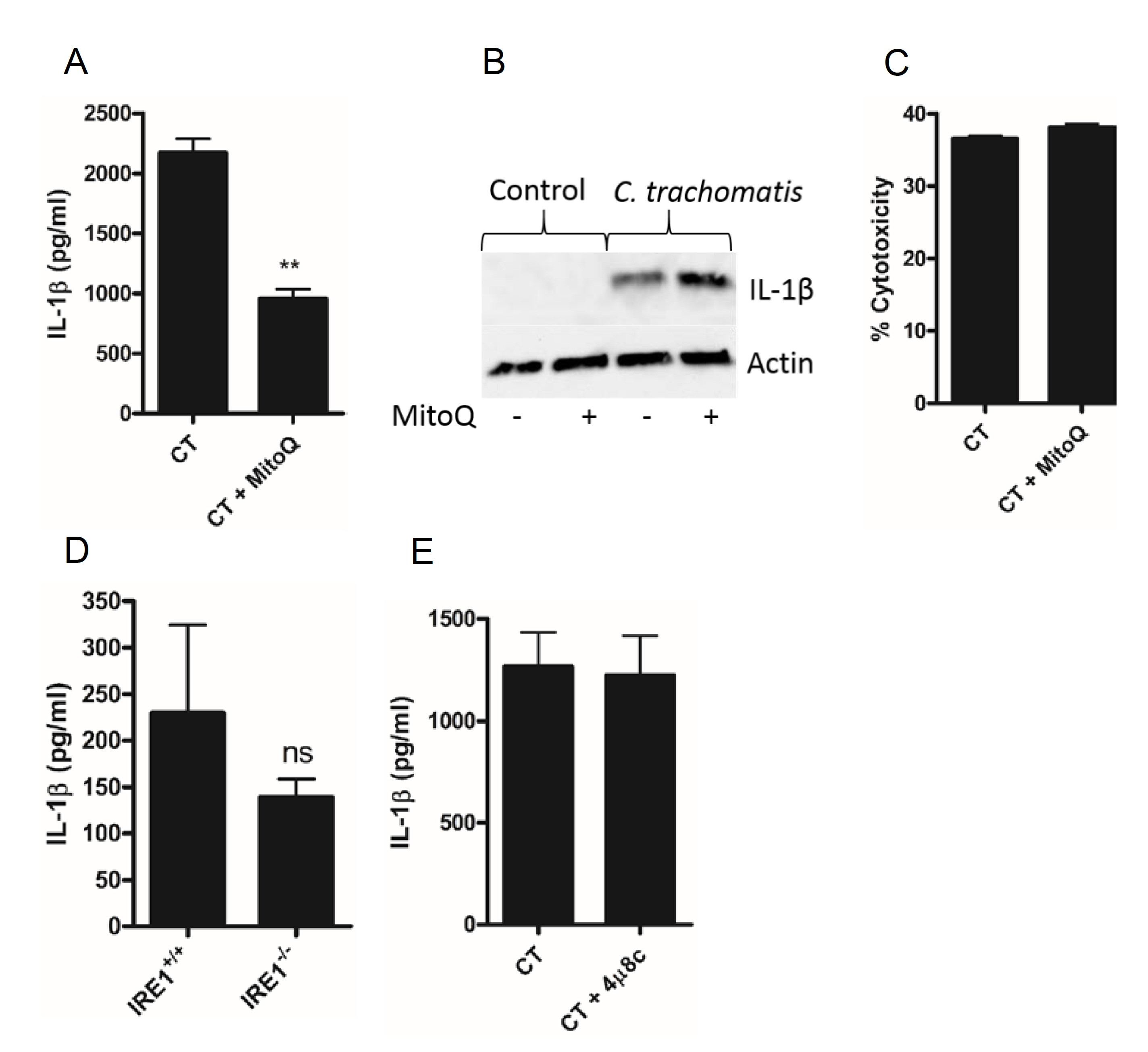

Supplement: S1 Fig — (A) IL-1β secretion analysed by ELISA of supernatants wild-type BMDM infected with C. trachomatis (CT) presence of the mitochondrial anti-oxidant; MitoQ (1μM) for 24hrs (B) Pro IL-1β expression analysed by western blotting of lysates from unstimulated or C. trachomatis infected BMDM in the presence (+) or absence (-) of 1μM MitoQ for 24hrs. (C) Cell death analysed by LDH release from wild-type BMDM infected with C. trachomatis (CT) presence of the mitochondrial anti-oxidant; MitoQ (1μM) for 24hrs. (D) IL-1β secretion analysed by ELISA of supernatants from C. trachomatis (CT) infected wild-type (IRE-1+/+) or IRE1 knock-out (IRE-1-/-) BMDM for 24hrs. (E) IL-1β secretion analysed by ELISA of supernatants from wild-type BMDM infected with C. trachomatis (CT) in the presence of the IRE1 inhibitor 4μ8c (30μM) for 24hrs. Data represented as the mean of one experiment performed on cells from three individual mice, error bars indicate ±SEM. *p = <0.05, **p = <0.01 and ***p = <0.001. ns indicates no statistical significance between samples. (TIF) [file ppat.1006383.s001.tif]

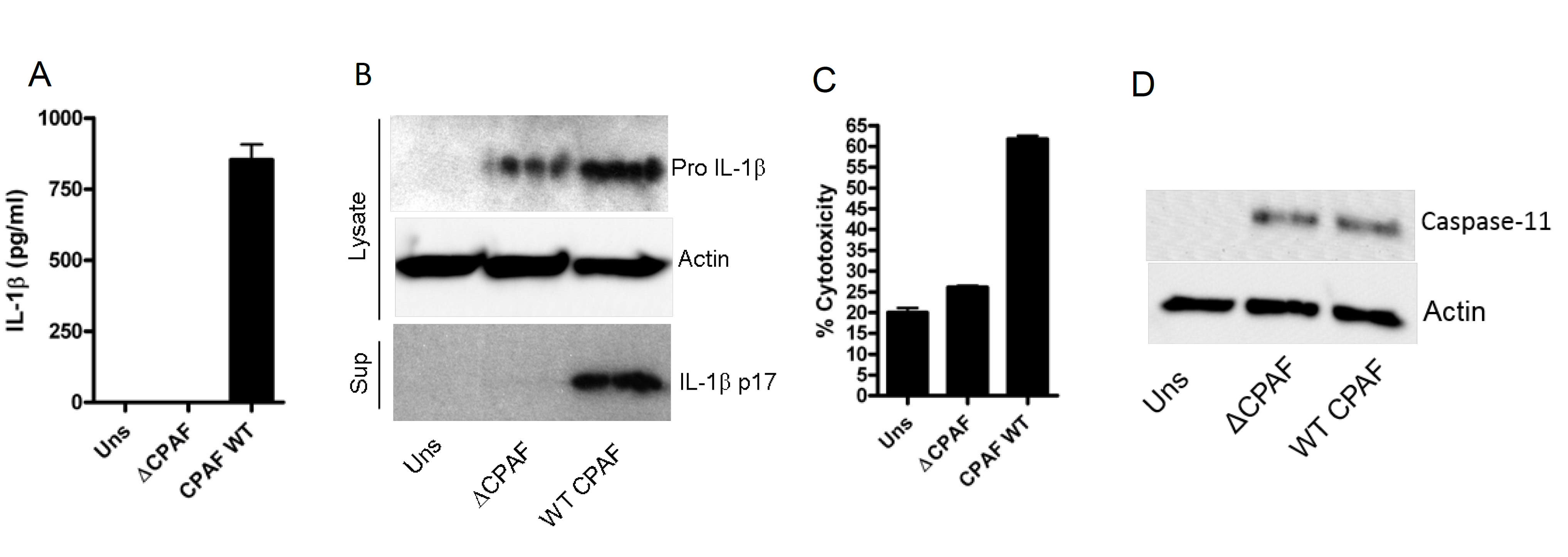

Supplement: S2 Fig — (A) IL-1β secretion analysed by ELISA of supernatants from wild-type BMDM infected with CPAF deficient (ΔCPAF) or CPAF sufficient control (CPAF WT) C. trachomatis. (B) IL-1β maturation and priming analysed by western blotting of cell lysates and supernatants from wild-type BMDM infected with CPAF deficient (ΔCPAF) or CPAF sufficient control (CPAF WT) C. trachomatis for 24hrs. (C) cell death analysed by LDH release from wild-type BMDM infected with CPAF deficient (ΔCPAF) or CPAF sufficient control (CPAF WT) C. trachomatis for 24hrs. Data represented as the mean of one experiment performed on cells from three individual mice, error bars indicate ±SEM. (D) Caspase-11 expression analysed by western blotting of lysates from BMDM infected with deficient (ΔCPAF) or CPAF sufficient control (CPAF WT) C. trachomatis for 24hrs. (TIF) [file ppat.1006383.s002.tif]

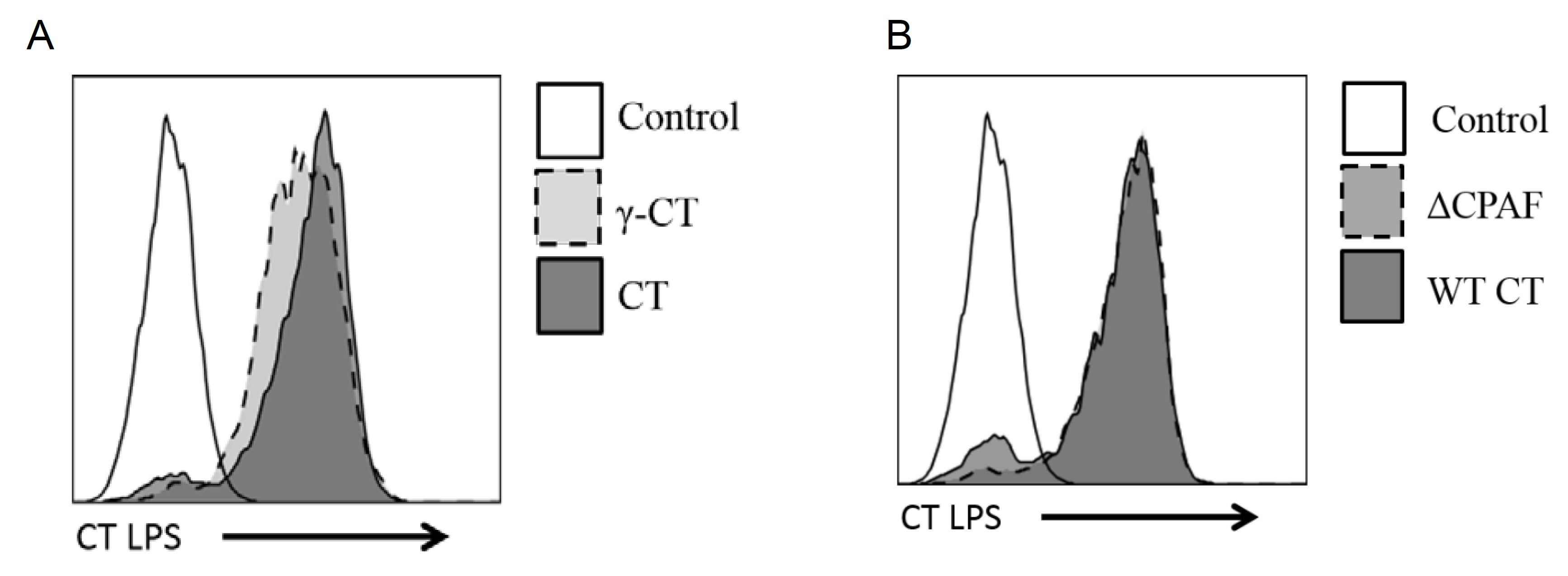

Supplement: S3 Fig — (A) Intracellular staining of C. trachomatis LPS in BMDM analysed by FACS following infection with irradiated C. trachomatis (γ-CT) or non-attenuated C. trachomatis (CT). (B) Intracellular staining of C. trachomatis LPS in BMDM analysed by FACS following infection with CPAF deficient C. trachomatis (ΔCPAF) or wild-type C. trachomatis (WT CT). (TIF) [file ppat.1006383.s003.tif]

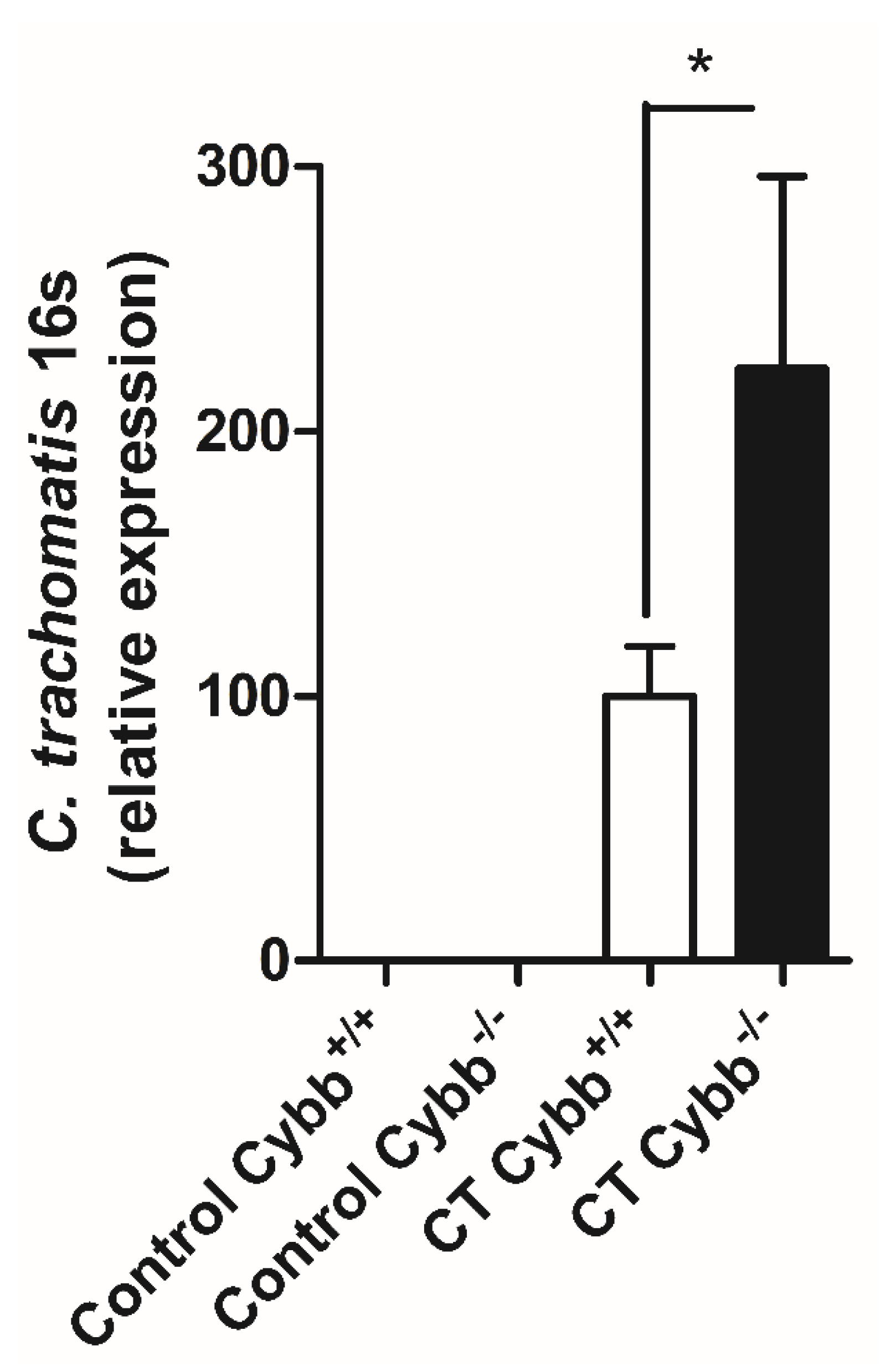

Supplement: S4 Fig — C. trachomatis replication in wild type (Cybb+/+) or Cybb deficient (Cybb-/-) BMDM analysed by qRT-PCR of C. trachomatis 16s RNA expression following C. trachomatis infection for 6-hours. Data represented as the mean of one experiment performed on BMDM from three individual mice, error bars indicate ±SEM *p = <0.05. (TIF) [file ppat.1006383.s004.tif]

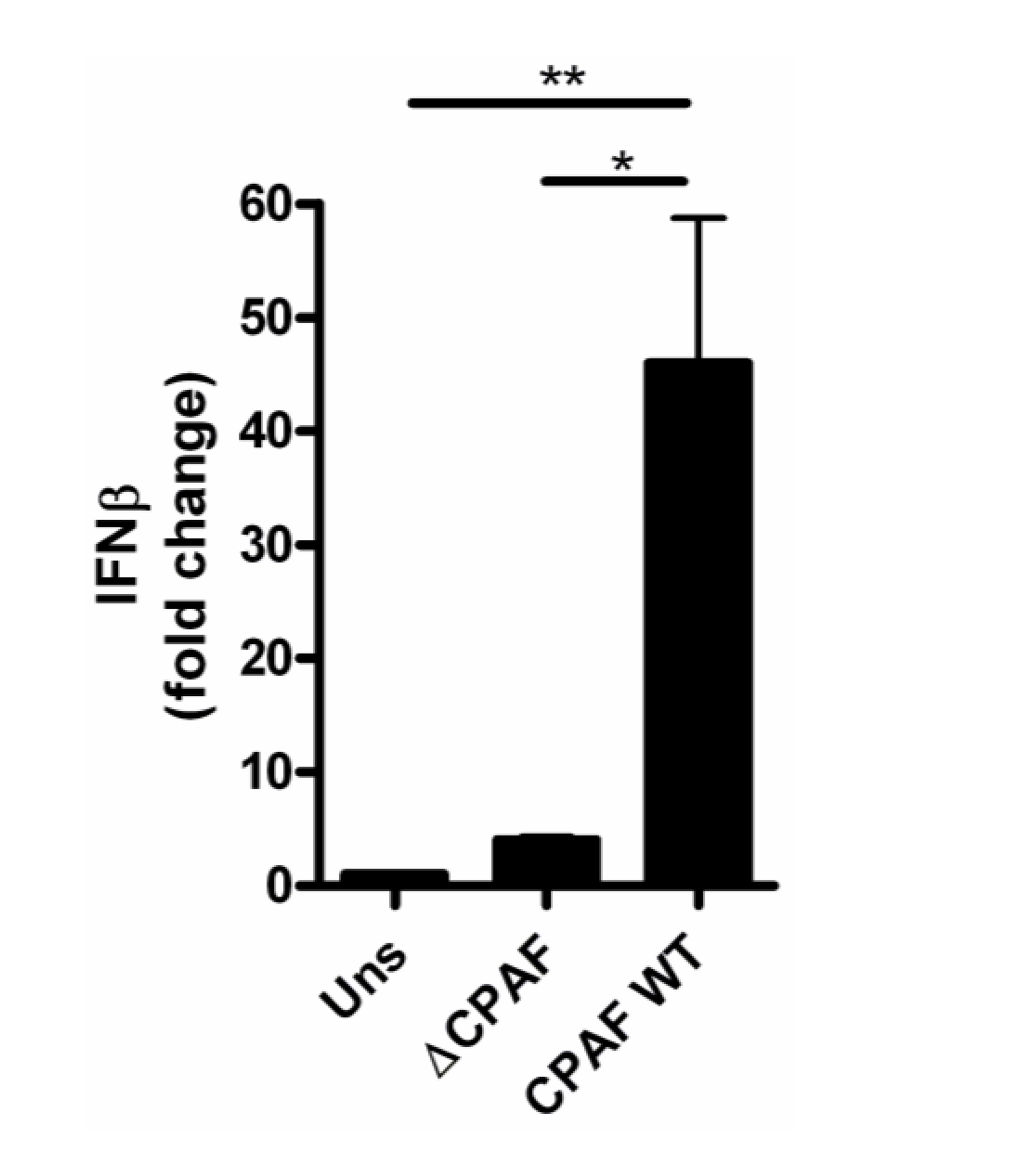

Supplement: S5 Fig — Induction of IFNβ mRNA expression in wild type BMDM analysed by quantitative RT-PCR following infection with CPAF deficient (ΔCPAF) or CPAF sufficient control (CPAF WT) C. trachomatis for 8hrs. Data represented as the mean of one experiment performed on cells from three individual mice, error bars indicate ±SEM. *p = <0.05, **p = <0.01. (TIF) [file ppat.1006383.s005.tif]
